# SGLT2 Inhibitors and Their Effect on Urolithiasis: Current Evidence and Future Directions

**DOI:** 10.3390/jcm13196017

**Published:** 2024-10-09

**Authors:** Živka Dika, Marijana Živko, Marina Kljajić, Bojan Jelaković

**Affiliations:** 1 School of Medicine, University of Zagreb, Šalata 3, 10000 Zagreb, Croatia; jelakovicbojan@gmail.com; 2 Department of Nephrology, Arterial Hypertension, Dialysis and Transplantation, University Hospital Center Zagreb, Kišpatićeva 12, 10000 Zagreb, Croatia; marijana.zivko3@gmail.com (M.Ž.); marina.kljajic7@gmail.com (M.K.)

**Keywords:** SGLT-2 inhibitors, urolithiasis, nephrolithiasis, kidney stone formers, epidemiology, diabetes mellitus

## Abstract

Urolithiasis (UL) is increasingly prevalent due to rising cardiorenometabolic diseases, posing significant management challenges despite advances in urological techniques. Sodium-glucose cotransporter-2 (SGLT2) inhibitors, primarily used for type 2 diabetes mellitus, chronic kidney disease, and heart failure, have emerged as a potential novel approach for UL treatment. These inhibitors may help reduce the risk of urolithiasis, particularly in patients with diabetes, by improving glycemic control and altering urinary chemistry, which are crucial factors in stone formation. However, the changes in urinary composition induced by SGLT2 inhibitors might also increase the risk of uric acid stone formation. This review evaluates the potential of SGLT2 inhibitors in managing UL, highlighting both the benefits and the risks. While these inhibitors show promise in reducing new and recurrent urinary stones in patients with diabetes, data on their effects in patients without diabetes who form stones are limited. Current human evidence largely comes from post hoc analyses of randomized controlled trials (RCTs) and large-scale database studies, with only one study providing detailed stone composition data. Experimental studies in animal models and cell lines have focused on calcium oxalate (CaOx) stones, showing that SGLT2 inhibitors specifically target CaOx stone formation and related renal inflammation. Although primarily studied for CaOx stones, their potential impact on other calcium-containing stones, such as calcium phosphate, remains promising. Further research is needed to explore their therapeutic potential and optimize treatment strategies.

## 1. Introduction

Urolithiasis (UL) refers to the formation of stones in the urinary tract. Over the past three decades, the global burden of UL has significantly increased, with both incidence and prevalence rising worldwide. These trends vary regionally, influenced by demographic, genetic, geographic, and environmental factors [1,2]. Worldwide, the incidence of urinary stones has risen by 48.57%, while urolithiasis-related disability-adjusted life years (DALYs) have increased by 16.95% over the last 30 years [1]. However, age-standardized incidence rates (ASIR) and age-standardized DALYs have decreased during this period. This trend indicates the overall rise in cases due to factors such as population growth and aging, while the decline in age-standardized rates suggests that advances in healthcare, earlier detection, and better management have mitigated the impact of the disease across different age groups [1]. The prevalence of UL has also increased substantially, with recent estimates ranging from 1.7% to 21.1%. This wide variation reflects differing rates across regions, with the highest prevalence observed in Asia’s “Stone Belt” region, which includes West Asia (including the Middle East), Southeast Asia, South Asia, South Korea, and Japan, where rates range from 5% to 21.1%. Notably, Southern Iran reports a prevalence of 21.1%, while Saudi Arabia and South Korea have rates of up to 19.1% and 11.5%, respectively. Comparable patterns are observed worldwide, particularly in the southern and southwestern United States (the “U.S. stone belt”) and parts of the Mediterranean in Europe. These trends are driven by factors such as high ambient temperatures, dehydration, oxalate-rich diets, and genetic predispositions, emphasizing the need for targeted prevention and treatment strategies [2,3,4,5,6].

Additionally, these rising trends are partially attributed to the growing incidence of cardiorenometabolic (CRM) diseases and lifestyle changes over time [1]. CRM conditions such as diabetes, hypertension, obesity, dyslipidemia, metabolic syndrome, and chronic kidney disease (CKD) are significant risk factors for UL. Conversely, the presence of urinary stones is also associated with an increased risk of developing these CRM conditions [2,7,8,9,10,11,12,13,14,15,16,17]. Furthermore, metabolic syndrome and its components are strongly associated with UL, with greater severity of stone disease observed when these factors are combined [7].

Interestingly, diabetes and obesity pose higher risks for stone formation in women than in men [17,18,19,20]. This discrepancy may be attributed to several factors. Women with diabetes and obesity may experience hormonal and metabolic changes that exacerbate the risk of stone formation. For instance, hormonal fluctuations and variations in fat distribution can influence calcium and oxalate metabolism, which are key components in stone formation [21]. Additionally, differences in dietary habits, body composition, and the impact of obesity-related comorbidities may contribute to the increased susceptibility in women [19,20,22]. Understanding these gender-specific differences is crucial for developing targeted prevention and treatment strategies for UL.

Given its multifactorial nature and bidirectional relationship with CRM diseases, UL should be considered a systemic disorder. Despite advancements in urological techniques such as minimally invasive surgery and enhanced lithotripsy, these methods focus primarily on the mechanical removal of stones and do not address the underlying pathophysiological processes contributing to UL. Similarly, current pharmacological treatments have limitations in targeting these core mechanisms [23,24].

Inhibitors of sodium-glucose cotransporter-2 (SGLT2), or gliflozins, are a newer class of antihyperglycemic drugs that inhibit renal glucose and sodium reabsorption at the S1 and S2 segments of the proximal tubule, thereby enhancing glucosuria and natriuria [25]. In addition to their role in glycemic control, these drugs offer multiple CRM benefits, including blood pressure reduction [26,27,28], body weight loss [29], improved renal and cardiovascular function, and decreased hospitalization and mortality [30,31,32,33,34,35,36,37] They also improve lipid profile and uricemia [38,39,40]. SGLT2 inhibitors improve cardiovascular and renal outcomes in patients with cardiovascular disease (CVD) and heart failure (HF), regardless of ejection fraction or the presence of type 2 diabetes (T2D). These drugs play an increasingly important role in managing HF and CKD beyond glucose control. By inhibiting glucose and sodium reabsorption, SGLT2 inhibitors reduce glomerular hydrostatic pressure and hyperfiltration, alleviating kidney stress and improving renal hemodynamics, even in patients with advanced CKD. This not only slows the decline in kidney function but also enhances renal protection in both patients with and without diabetes. Their diuretic and natriuretic effects lower blood pressure, reduce left ventricular preload and afterload and improve heart failure outcomes. Additionally, SGLT2 inhibitors mitigate hyperkalemia, arrhythmias, and left ventricular dysfunction while reducing sympathetic tone and uric acid levels. They are also demonstrated to have anti-inflammatory effects, which may further enhance vascular health and kidney function, contributing to reduced cardiovascular events and slowed progression of kidney disease. These benefits manifest early and are independent of glucose-lowering effects, underscoring the broader role of SGLT2 inhibitors in cardiorenal protection [30,31,32,33,34,35,36,37,41].

Emerging evidence suggests that SGLT2 inhibitors may offer a novel therapeutic approach by influencing the metabolic pathways involved in stone formation. These inhibitors have been shown to reduce the incidence of both new and recurrent urinary stones, although they may also increase the risk of uric acid stone formation (Table 1) [42,43]. Therefore, further research is necessary to fully elucidate these effects and refine management strategies for UL.

In this review, we examine the impact of SGLT2 inhibitors on urinary stone formation and assess their potential as a therapeutic option for UL based on the current evidence.

## 2. Mechanism of SGLT-2 Inhibitors and Its Role in Urinary Stone Formation: Risks and Benefits

Recent studies suggest that SGLT2 inhibitors may reduce the risk of urinary stone formation, demonstrating their complex pharmacological effects beyond their initial use for diabetes management, as well as their roles in HF and CKD [30,31,32,33,34,35,36,37]. This section examines the mechanisms by which SGLT2 inhibitors influence urinary stone formation, considering both the potential risks and the therapeutic benefits.

### 2.1. Risks of Urinary Stone Formation

#### 2.1.1. Glycosuria, Increased Solute Load and Volume Depletion

SGLT-2 inhibitors increase glycosuria, altering urinary composition through osmotic diuresis. This diuresis initially increases urine output, diluting stone-forming solutes like calcium, oxalate, and uric acid. However, if diuresis is prolonged and fluid intake is insufficient, it may lead to dehydration and volume depletion, which in turn concentrates these solutes and increases the risk of calcium oxalate and uric acid stone formation [44,45]. The risk of dehydration counteracts the protective effect of dilution, creating a complex risk–benefit scenario for stone formation [35].

#### 2.1.2. Hypercalciuria and Calcium Stone Formation

SGLT-2 inhibitors can lead to increased calcium excretion (hypercalciuria) by inhibiting sodium reabsorption in the proximal tubules. This, combined with dehydration and reduced urine volume, enhances the concentration of calcium and the risk of forming calcium-based stones. Although diuresis can dilute solutes, the concurrent risk of volume depletion exacerbates stone formation in susceptible patients [46,47].

#### 2.1.3. Altered Urinary pH

The glycosuria induced by SGLT-2 inhibitors can affect urinary pH through multiple mechanisms. Elevated glucose levels provide a substrate for bacterial metabolism, leading to the production of organic acids, such as lactic and acetic acids, which lower urinary pH and increase the risk of uric acid stone formation. Patients with diabetes are particularly susceptible to urinary tract infections (UTIs) due to their increased glycosuria, which fosters bacterial growth and complicates urinary pH dynamics. Infections involving urease-producing bacteria can hydrolyze urea into ammonia, raising urinary pH and promoting struvite stone formation. Additionally, SGLT-2 inhibitors may influence urinary pH through osmotic diuresis and altered bicarbonate handling, further affecting stone risk dynamics [47,48].

In summary, the interplay of glycosuria, bacterial metabolism, and SGLT-2 inhibitors creates a complex environment for urinary pH regulation. This interaction can lead to dual stone formation risks: a lower urinary pH increases the likelihood of uric acid stones, while a higher pH—often due to urease-producing bacteria—can promote struvite stones. Understanding these dynamics is crucial for managing patients on SGLT-2 inhibitors, as the specific bacterial flora and underlying conditions significantly influence stone risk.

#### 2.1.4. Effects on Oxalate Metabolism

Although not fully understood, SGLT-2 inhibitors may impact oxalate metabolism by increasing hypercalciuria, which can indirectly elevate urinary oxalate excretion and enhance the risk of calcium oxalate stone formation. Additionally, by altering microbial composition, these inhibitors may promote the growth of oxalate-degrading bacteria, potentially reducing oxalate absorption and improving gut health. While evidence remains limited, these interactions could play a role in managing oxalate levels and reducing the risk of urinary stone formation [49].

#### 2.1.5. Uricosuria and Uric Acid Stone Formation

SGLT-2 inhibitors increase uric acid excretion, which may elevate the risk of uric acid stones, particularly in acidic urine. However, by promoting a higher urinary pH, these drugs can also reduce the likelihood of uric acid stone formation by keeping uric acid in a more soluble form [50]. This dual effect underscores the need for close monitoring of urinary pH in patients at risk for uric acid stones.

### 2.2. Protective Benefits against Urinary Stone Formation

#### 2.2.1. Increased Urinary Citrate Excretion

SGLT-2 inhibitors enhance urinary citrate excretion by modulating renal sodium and bicarbonate handling. Citrate binds to calcium in the urine, reducing its availability for stone formation, particularly calcium oxalate and calcium phosphate stones. Additionally, increased citrate excretion promotes a more alkaline urinary environment, reducing the risk of uric acid stones [51]. Although an alkaline pH can increase the risk of calcium phosphate stones, citrate’s ability to bind calcium mitigates this risk [52].

#### 2.2.2. Reduction in Uricemia and Uric Acid Stone Formation

SGLT-2 inhibitors promote uricosuria while lowering serum uric acid levels, decreasing the overall risk of uric acid stones. Despite elevated urinary uric acid levels, the lower systemic uric acid concentration reduces the driving force for uric acid precipitation and stone formation [50].

#### 2.2.3. Osmotic Diuresis and Dilution of Solutes

The diuretic effect of SGLT-2 inhibitors increases urine volume, leading to the dilution of stone-forming solutes like calcium, oxalate, and uric acid. This dilution reduces the likelihood of crystal formation and stone development. However, careful management of fluid intake is crucial to prevent dehydration, which can negate the protective effect of concentrating solutes [35].

#### 2.2.4. Weight Loss and Reduction in Risk Factors for Stone Formation

SGLT-2 inhibitors also facilitate modest weight loss, which reduces the risk of UL, particularly in individuals with metabolic syndrome or obesity. Weight loss improves insulin sensitivity, reduces urinary calcium and oxalate excretion, and increases urinary citrate levels, all of which help reduce the likelihood of stone formation. In addition, by lowering the risk of hypertension and diabetes, SGLT-2 inhibitors indirectly mitigate stone formation risk [53].

### 2.3. Conclusions

SGLT-2 inhibitors present both risks and protective benefits in relation to urinary stone formation. They increase the excretion of calcium, oxalate, and uric acid and influence urinary pH, all of which can elevate the risk of calcium and uric acid stones. On the other hand, these drugs enhance urinary citrate excretion, reduce serum uric acid levels, promote diuresis that dilutes stone-forming solutes, and assist with weight loss, which collectively help lower stone risk. Proper hydration and careful monitoring of urinary pH and calcium levels are critical in managing these risks, particularly for patients at high risk of stone formation. Further research is needed to better understand which populations will benefit the most from SGLT-2 inhibitors while minimizing the risk of UL.

## 3. Clinical Research on the Effects of SGLT2 Inhibitors in Urolithiasis: Evidence from Human Populations with and without Diabetes and Experimental Models

### 3.1. Data on SGLT2 Inhibitors Compared to Placebo and/or Other Glucose-Lowering Agents in Human Populations—Evidence from Patients with and without Diabetes

In a 2015 post hoc analysis of pooled data from four multinational, randomized trials, Davies and colleagues found no significant difference in nephrolithiasis incidence between patients with type 2 diabetes (T2D) treated with canagliflozin and those receiving a placebo [54]. Only one case of nephrolithiasis was reported, occurring in the placebo group without baseline hyperuricemia (Table 1). However, a meta-analysis of randomized placebo-controlled trials involving 15,081 patients with T2D assigned to either empagliflozin or placebo showed a reduced risk of nephrolithiasis as a reported adverse event with empagliflozin [55]. This analysis defined nephrolithiasis using both broad and narrow criteria. The broad definition included MedRA terms such as nephrolithiasis, renal colic, ureterolithiasis, bladder calculus, urinary calculus, urethral calculus, and nephrocalcinosis, while the narrow definition, used in a sensitivity analysis, excluded renal colic and nephrocalcinosis. During the follow-up period, 183 patients developed UL, with 79 cases in the placebo group and 104 in the empagliflozin group. This resulted in annual incidence rates of 1.01 events per 100 patient-years for the placebo group and 0.63 events per 100 patient-years for the empagliflozin group. The incidence rate ratio (IRR) was 0.64 (95% CI, 0.48–0.86), indicating a lower risk with empagliflozin. A sensitivity analysis using the narrower definition confirmed similar results, with an IRR of 0.62 (95% CI, 0.45–0.85) in favor of empagliflozin (Table 1). Additionally, Anan and colleagues, in a recent cross-sectional population study using administrative database data, found a reduced risk of UL—defined as kidney and ureter stones—in men with T2D treated with SGLT2 inhibitors [41]. The study revealed that the prevalence of UL was consistently higher in men compared to women, regardless of SGLT2 inhibitor treatment. Specifically, the prevalence of UL was 2.38% in men compared to 1.58% in women among patients with diabetes treated with SGLT2 inhibitors and 2.54% in men compared to 1.66% in women among patients with diabetes not treated with these inhibitors. The majority of UL cases documented in the study were in the 60–79 age group for both genders. These findings align with the higher prevalence of UL in men and the increasing trend of UL with age observed in the general population [2]. In the study, the odds ratio (OR) for men across all age groups was 0.89 (95% CI: 0.86–0.94), indicating a lower risk compared to patients with T2D not treated with SGLT2 inhibitors, with a significant reduction in risk observed across most age groups except for those aged 80 and above. In contrast, no similar reduction in risk was observed in women (OR for women across all age groups = 0.95, 95% CI: 0.89–1.02) (Table 1). However, it is important to consider the study’s limitations. The study’s retrospective design and the lack of data on several important factors, such as kidney function, metabolic profile (including lipid and lithogenic profiles), stone composition, BMI, diet, and comorbidities like arterial hypertension, heart disease, and kidney disease, may affect the interpretation and generalizability of the findings Additionally, the study population was obtained from a Japanese database, which may limit the applicability of the results to other populations due to potential ethnic and regional differences in health profiles and medical practices.

Several studies have investigated the impact of SGLT2 inhibitors on UL compared to other glucose-lowering agents in the population with diabetes. In a recent post hoc analysis of 16 randomized controlled clinical trials (RCTs) involving patients with T2D, Cosentino and colleagues examined the incidence of nephrolithiasis as a serious adverse event reported by investigators [56]. The meta-analysis compared the effects of SGLT2 inhibitors (canagliflozin, dapagliflozin, empagliflozin, and ertugliflozin) with either a placebo or other antidiabetic drugs (insulin secretagogues and metformin). Nephrolithiasis was documented in only 62 cases among the 28,052 patients treated with SGLT2 inhibitors, compared to 44 cases in the control group of 19,623 patients. The authors found no association between SGLT2 inhibitors and nephrolithiasis, with a Mantel–Haenszel odds ratio (MH-OR) of 0.85 (0.57–1.26). In addition, they found no difference in the risk of nephrolithiasis among the SGLT2 inhibitors in the study nor in the risk of nephrolithiasis between SGLT2 inhibitors and other drug comparators (Table 1). On the other hand, Kristensen and colleagues found a significantly lower rate of both incident and recurrent urolithiasis among patients with diabetes initiating SGLT2 inhibitors compared to those using glucagon-like peptide-1 receptor agonists (GLP-1 RAs) [43]. In their nationwide observational retrospective cohort study, the hazard ratios were 0.51 (95% CI 0.37–0.71) for incident nephrolithiasis and 0.68 (95% CI 0.48–0.97) for recurrent nephrolithiasis. Additionally, the authors found a significantly lower risk of incident nephrolithiasis among patients with diabetes using SGLT2 inhibitors compared to those using dipeptidyl peptidase-4 (DPP-4) inhibitors (gliptins) in the same study, with a hazard ratio of 0.61 (95% CI 0.41–0.88). When examining the SGLT2 inhibitors separately, the results remained consistent: the hazard ratio was 0.55 (95% CI 0.36–0.86) for empagliflozin and 0.56 (95% CI 0.39–0.79) for dapagliflozin, both compared to GLP-1 RAs (Table 1). Despite several limitations, this study offers more accurate risk estimates of nephrolithiasis associated with SGLT2 inhibitors in a routine clinical setting compared to RCTs. Nationwide databases often provide a more comprehensive view of real-world clinical settings because they encompass a broader and more diverse patient population. In contrast, RCTs typically involve stricter exclusion criteria and a narrower patient group, which can limit the generalizability of their findings.

Further supporting the previous beneficial effects, Anan and colleagues [20] investigated the associations between various oral glucose-lowering drug classes—including α-glucosidase inhibitors, biguanides, DPP-4 inhibitors, glinides, SGLT2 inhibitors, sulfonylureas, thiazolidinediones, and combination drugs—and urolithiasis in patients with diabetes. They found that only SGLT2 inhibitors (such as canagliflozin, dapagliflozin, empagliflozin, ipragliflozin, luseogliflozin, tofogliflozin, and fixed-dose combinations of SGLT2 inhibitors and gliptins) were negatively associated with the new onset of UL in patients with diabetes, compared to those not using these medications (OR for men = 0.95; 95% CI, 0.91–0.98 and OR for women = 0.91; 95% CI, 0.86–0.97). In contrast, other glucose-lowering drugs, including α-glucosidase inhibitors, biguanides, DPP-4 inhibitors, glinides, sulfonylureas, thiazolidinediones, and their combinations, were significantly associated with an increased risk of UL in both men and women. Additionally, the study showed that among patients without diabetes, the use of SGLT2 inhibitors was significantly associated with a lower likelihood of developing UL, though this effect was observed only in men (OR for men = 0.42; 95% CI, 0.35–0.51; OR for women = 0.90; 95% CI, 0.68–1.19) (Table 1). However, it is important to note that the patients without diabetes included in this analysis were primarily those with HF and/or CKD, as several SGLT2 inhibitors are indicated for these conditions in Japan, whose national health database was used for the analysis. This presents a potential limitation due to selection bias, which may restrict the generalizability of the findings to patients without diabetes without these specific conditions. Additionally, the authors could not examine other risk factors for UL, such as hypertension and obesity, due to limitations in the database. While this study demonstrated a significant reduction in the risk of UL with SGLT2 inhibitor use in men without diabetes, the effect in women without diabetes was not statistically significant. Nevertheless, these findings, along with evidence from previous studies, suggest that SGLT2 inhibitors significantly reduce the risk of both incident and recurrent UL, particularly in individuals with diabetes, who have a high prevalence of this condition. This makes SGLT2 inhibitors a compelling option as the preferred glucose-lowering therapy in this high-risk population, who are especially vulnerable to the recurrence of stone formation and complications related to UL, including UTIs. However, it is important to note that SGLT2 inhibitors are generally associated with an increased risk of UTIs, which should be carefully evaluated when considering the overall benefit-risk profile of these medications for individual patients.

Unlike previous studies, only one has specifically examined the effect of SGLT2 inhibitors on urinary lithogenic profiles. In a single-center, double-blind, randomized, placebo-controlled trial, Harmacek and colleagues investigated the impact of four weeks of 10 mg empagliflozin treatment on urinary risk factors for stone formation in healthy volunteers [57]. Participants were randomized to receive either empagliflozin or a placebo, with split 24 h urine collections (daytime and nighttime) conducted at baseline and after one month. After four weeks, empagliflozin treatment led to a 45.45% increase in citrate excretion and a significant rise in urinary glucose excretion, while both diurnal and nocturnal urinary pH levels decreased. There were small but significant increases in hemoglobin, indicating mild volume contraction, though hematocrit changes were not significant (*p* = 0.06). Plasma electrolytes, creatinine, and urea levels remained stable, and there were no changes in 24 h urinary electrolyte excretion. Both the empagliflozin and placebo groups showed similar increases in 24 h urine volume, likely due to the hydration protocol. Empagliflozin also caused a significant reduction in plasma uric acid levels (86 ± 36 mmol/L) and increased uricosuria, both diurnally and nocturnally (Table 1). The relative supersaturation ratio (RSR) for calcium phosphate minerals (brushite and hydroxyapatite) decreased in the empagliflozin group, while the RSR for uric acid increased. In contrast, the placebo group experienced a decrease in the RSR for uric acid, with no significant changes in the RSR for calcium oxalate in either group. Overall, empagliflozin significantly altered key parameters associated with urinary lithogenic risk compared to placebo, including increased citrate excretion and reduced urinary pH. Although empagliflozin treatment was associated with an increase in uricosuria, this change was modest and did not reach statistical significance. These findings suggest that while empagliflozin may lower the risk of calcium phosphate (CaP) stone formation—due to decreased CaP supersaturation from reduced urinary pH and increased citrate—it might concurrently raise the risk of uric acid stones because of increased uricosuria and lower urinary pH. These changes in urinary chemistry may influence the formation of Randall’s plaque, a mineral deposit on renal papillae composed of calcium and magnesium phosphates [58]. This plaque acts as a nidus (core) for stone formation, particularly for calcium oxalate (CaOx) stones, which are the most common type of kidney stones [24]. Although Randall’s plaque is often found in individuals who do not develop kidney stones, its presence alone is not sufficient for stone formation [58]. Additional factors are needed to contribute to kidney stone development [2,42,59]. Thus, while empagliflozin may potentially reduce the risk of calcium-containing nephrolithiasis by lowering calcium phosphate supersaturation, careful monitoring for increased uric acid stone risk is essential. This highlights the complexity of stone formation beyond Randall’s plaque, emphasizing the importance of further research in stone-forming patients, both with and without diabetes, to better determine the role of this drug class in the management and prevention of urinary stones.

An ongoing study, the SWEETSTONE trial, is examining this issue further by exploring the effects of empagliflozin on urinary supersaturations in people without diabetes who form kidney stones [60]. Building on existing research findings (Table 1), this single-center, randomized, double-blind, placebo-controlled, cross-over study will assess whether empagliflozin can effectively reduce the risk of nephrolithiasis recurrence by influencing urinary supersaturations of CaOx, CaP, and uric acid. The trial includes 46 participants with a history of nephrolithiasis and specific stone compositions, aiming to generate crucial preliminary data on the drug’s prophylactic potential. If successful, the findings could shape future management strategies for UL by offering a novel approach to recurrence prevention. However, the study also emphasizes the need for careful monitoring of uric acid stone risk, providing insights into both the therapeutic benefits and the complexities of managing stone formation.

Table 1 summarizes the data on the type of study, study population, follow-up period, main findings, and limitations of the discussed studies.

**Table 1:** Studies on the effect of SGLT2 inhibitors on urolithiasis in human population.

Study (First Author and Year)	Type of Study	Study Population	Main Findings	**Limitations**
SGLT2Is vs. placebo in population with diabetes				
Davies et al. (2015) [54]	Cohort study utilizing data from 4 randomized, phase III, multinational trials FU 26 weeks	Pts with T2D Overall cohort, *n* = 2313 Cohort with HU, *n* = 115 Pts from both cohorts divided into 3 treatment groups—canagliflozin 100 mg, canagliflozin 300 mg, and placebo.	The incidence rates of NL similar between treatment groups in the overall pooled cohort. No difference in incidence of NL between treatment groups in the cohort with HU.	Post hoc analysis of pooled data 1 pt with reported NL in the placebo group in overall cohort No data on metabolic profile (lipid, lithogenic profile), stone composition, diet, and comorbidities available.
Balasubramanian et al. (2022) [55]	Meta-analysis of 20 RCTs phase I–IV Median FU for placebo = 543 days and FU for empagliflozin = 549 days	Pts with T2D *n* = 15,081 Pts divided into 2 treatment groups: empagliflozin (10 mg or 25 mg) *n* = 10,177, and placebo *n* = 4904	Empagliflozin therapy is associated with a 36% reduction in the risk of UL events in patients with T2D compared to placebo, with annual incidence rates of 0.63 vs. 1.01 events per 100 PY. The IRR is 0.64 (95% CI, 0.48–0.86) using a broad definition, and 0.62 (95% CI, 0.45–0.85), indicating a 38% risk reduction in a narrower sensitivity analysis.	Post hoc analysis of pooled data No data on metabolic profile (lipid, lithogenic profile), stone composition, diet available.
SGLT2Is vs. other glucose-lowering agents in population with diabetes				
Anan et al. (2022) [42]	A cross-sectional nationwide population study utilizing data from an administrative database	Pts with DM aged ≥ 20 *n* = 1,538,198 (men, *n* = 909,628) Two study groups: SGLT2i-treated and non-SGLT2i treated group Included pts with DM type 1 and 2 based on ICD-10 codes	Prevalence of NL higher in men compared to women regardless of SGLT2i treatment (2.38% vs. 1.58% on SGLT2i; 2.54% vs. 1.66% not on SGLT2i). Risk of NL reduced only in men treated with SGLT2i (OR for men across all age groups = 0.89, 95% CI: 0.86–0.94), but not in women (OR for women across all age groups = 0.95, 95% CI: 0.89–1.02). The reduced risk of NL in men treated with SGLT2i significant in all stratified age groups except for those aged ≥ 80.	No data on kidney function, metabolic profile (lipid, lithogenic profile), stone composition, BMI, diet and comorbidities available.
Cosentino et al. (2019) [56]	Meta-analysis of 27 RCTs FU ≥ 52 weeks (52–218 weeks)	Pts with T2D *n* = 28,052 on SGLT2i *n* = 19,623 on other comparators (placebo or active comparators including metformin, SU—glimepiride, glipizide; GLP1-RA—exenatide LAR; DPP—4i—sitagliptin, linagliptin) NL cases: 62 pts on SGLT-2i and 44 pts in control (comparator) group	No association between SGLT2Is and NL (MH-OR = 0.85 (0.57–1.26)); No difference in the risk of NL in SGLT2i subanalysis: OR for canagliflozin = 1.04 (CI 0.51–2.13), OR for dapagliflozin = 0.70 (CI 0.35–1.41), OR for empagliflozin = 0.82 (CI 0.43–1.60), and OR for ertugliflozin = 1.48 (CI 0.06–36.34). No difference in the risk of NL between SGLT2i and other comparators—risk of NL comparing SGLT-2i with different comparators: vs. insulin secretagogues OR = 0.69 (CI 0.17–2.79), vs. placebo OR = 0.87 (CI 0.57–1.33), vs. metformin OR = 1.17 (CI 0.06–24.66).	Post hoc analysis of 16 RCTs with reported case of NL as a SAE No demographic data other than mean age, laboratory data other than mean HbA1c available No data on renal function, comorbidities, stone composition, diet available.
Kristensen et al. (2021) [43]	A nationwide retrospective cohort study; data from administrative database of Danish health registries Median FU after matching = 2.0 years	Pts with DM ≥40 years without a history of NL when initiating the study medication Total study population *n* = 43,866 SGLT2Is cohort *n* = 24,290 GLP1 RAs cohort *n* = 19,576 After propensity score matching *: Matched patient pairs (SGLT2i and GLP1 RA) *n* = 12,325 Matched patient pairs (SGLT2i and GLP1 RA) for recurrent NL *n* = 731 Matched patient pairs (SGLT2i and DPP4I) *n* = 10,908	Initiation of SGLT2 inhibitors is associated with a 49% reduced risk of incident NL and a 32% reduced risk of recurrent NL compared to GLP-1 RAs (HR = 0.51 (95% CI 0.37, 0.71) for incident and 0.68 (95% CI 0.48–0.97) for recurrent NL). When compared to DPP-4 inhibitors, the risk of NL is reduced by 39% (HR = 0.61 (95% CI 0.41–0.88)). Specifically, empagliflozin and dapagliflozin show a 45% and 44% lower risk of NL, respectively, compared to GLP-1 RAs (HR = 0.55 (CI 0.36–0.86) for empagliflozin; HR = 0.56 (0.39–0.79) for dapafliflozin).	Type of DM not specified NL definition: based on diagnosis ICD-10 codes of N20 and N132 documented in the Danish National Patient Registry Use of SGLT2Is: Included both monotherapy formulations and fixed-dose combinations No laboratory data available No data on BMI, stone composition, diet available.
Anan et al. (2023) [20]	A cross-sectional nationwide population study utilizing data from an administrative database	Pts aged ≥ 20 Total study population, *n* = 11,931,480 Pts with DM, *n* = 1,746,992 (men, *n* = 1,026,574) Pts with DM and UL, *n* = 37,619 (men, *n* = 25,707) Pts with diabetes stratified by antidiabetic drug class and UL status	Women with DM have greater risk for developing UL than men (OR = 1.12 (95% CI 1.10–1.13) for male and OR = 1.70 (95% CI = 1.67–1.73) for female). Among various oral antidiabetic drug classes: SGLT2Is: Significant negative association with UL (OR for males = 0.95 (95% CI, 0.91–0.98), OR for females = 0.91 (95% CI, 0.86–0.97)); Other drug classes (α-glucosidase inhibitors, biguanides, DPP-4 inhibitors, glinides, sulfonylureas, thiazolidinediones): Significant positive association with UL. For details, see Table 2 of the reference [20].	No demographic data (other than gender) available. No laboratory data available No data on comorbidities, stone composition, diet, or use of other drugs (besides antidiabetic medications) available.
Population without diabetes on SGLT2Is				
Anan et al. (2023) [20]	A cross-sectional nationwide population study utilizing data from an administrative database	Pts aged ≥ 20 Total study population, *n* = 10,195,626 (men, *n* = 4,685,179) Pts on SGLT2i, *n* = 16,522 (men, *n* = 10,972) Pts on SGLT2i with UL, *n* = 155 (men, *n* = 106)	Among patients without diabetes prescribed SGLT2Is, there is a significant 58% reduced likelihood of developing UL in men (OR = 0.42; 95% CI, 0.35–0.51), but now in women (OR = 0.90; 95% CI, 0.68–1.19).	Patients without on SGLT2Is included those with HF and CKD No demographic data (other than gender) available. No laboratory data available No data on comorbidities, stone composition, diet, or use of other drugs (besides antidiabetic medications) available.
SGLT2Is vs. placebo in healthy volunteers				
Harmacek et al. (2022) [57]	A monocentric, double-blind, randomized, placebo-controlled study FU = 4 weeks	Healthy volunteers, *n* = 40 Participants randomized to placebo (*n* = 13) or empagliflozin group (*n* = 27)	Empagliflozin vs. Placebo: 24-h urine volume: Both groups experienced a similar increase in urine volume over the FU period without significant difference between the groups; Plasma electrolytes, creatinine, and urea: No significant differences between the groups observed. Urinary citrate excretion: Increased significantly in the empagliflozin by 45.45%, but not in the placebo group. Urinary pH levels: a significant decrease in both diurnal and nocturnal urinary pH levels in empagliflozin group, whereas no significant changes in the placebo group. SUA and uricosuria: significant reductions in SUA and insignificant increases in uricosuria in empagliflozin group, while no significant changes in either parameter in the placebo group. Relative supersaturation ratio (RSR) of minerals: Empagliflozin did not affect the RSR for CaOx, similar to the placebo group; The RSR for CaP minerals decreased in the empagliflozin group but not in the placebo group. A decrease in the RSR for uric acid in the placebo group and an increase in this ratio in empagliflozin group	Post hoc analysis.

* For details, see the Methods in reference [43] (Kristensen et al. (2021). BMI—Body mass index, CaOx- Calcium Oxalate, CaP—Calcium Phosphate, CI—Confidence Interval, CKD- Chronic kidney disease, DM—Diabetes mellitus, DPP-4i—Dipeptidyl peptidase-4 inhibitor (gliptin), eGFR—estimated glomerular filtration rate, FU—Follow up, GLP1-RA—Glucagon-like peptide-1 receptor agonists (Exenatide LAR), Hb—Hemoglobin, HbA1c—Glycated hemoglobin, HF—Heart failure, HU—Hyperuricemia, ICD-10—The International Classification of Diseases, 10th revision, IRR—Incidence rate ratios, MH-OR—Mantel–Haenzel Odds Ratio, NL—Nephrolithiasis, OR—Odds Ratio, Pts—Patients, PY—Patient years, RCT(s)—Randomized control trial(s), SGLT2i—Sodium-glucose co-transporter 2 inhibitor, SGLT2Is—Sodium-glucose co-transporter 2 inhibitors, SU—Sulfonylurea, SUA—Serum uric acid level, T2D—type 2 diabetes, UL—urolithiasis.

### 3.2. Data on Experimental Models of Urolithiasis and SGLT2 Inhibitors—Insights from Preclinical Research

Recent research highlights that Randall’s plaques, initial sites for kidney stone formation, are rich in calcium-binding proteins, notably osteopontin (OPN) [61]. OPN, produced by renal tubular cells, plays a crucial role in regulating CaOx crystal growth and aggregation, essential steps in CaOx kidney stone development [42,62,63]. Additionally, OPN acts as a proinflammatory cytokine, contributing to macrophage infiltration and interstitial fibrosis in kidney tissues [42,62,63,64,65,66].

Two main hypotheses exist regarding OPN’s role in stone formation: it may act as either an inducer or an inhibitor. Some studies suggest that OPN promotes stone formation [42,62,63,66,67], while others propose it inhibits stone formation [68,69]. Recent research by Anan and colleagues supports the hypothesis that OPN acts as an inducer [42]. Their findings indicate that SGLT2 inhibition reduces OPN production by interfering with glucose uptake in damaged proximal tubular epithelial cells, shifting the metabolic pathway toward glycolysis.

In experimental models, SGLT2 inhibitors have shown significant impacts on UL. In animal studies, ethylene glycol-induced CaOx kidney stones in rats were treated with phlorizin, an SGLT1/2 inhibitor. Phlorizin reduced CaOx stone formation, inflammation, and fibrosis by downregulating OPN and other injury markers, including CD44 [42]. CD44, a cell surface glycoprotein involved in cell adhesion and inflammation, plays a significant role in kidney stone formation by facilitating crystal attachment to renal cells. By modulating CD44 expression, SGLT inhibitors may reduce CaOx crystal adhesion, aiding in the prevention and management of UL. Phlorizin also attenuated interstitial fibrosis around infiltrated macrophages in CaOx stone-forming kidneys.

Similarly, SGLT2-deficient mice demonstrated resistance to glyoxylic acid-induced CaOx stone formation [42]. These mice exhibited decreased expression of OPN, CD44, transforming growth factor β1 (TGFβ1), fibronectin-1 (Fn1), and Alpha-Smooth Muscle Actin 2 (ACTA2), a marker for fibrosis and myofibroblast activation. This resulted in reduced inflammation and fibrosis in the kidneys. Immunostaining confirmed lower levels of OPN, FN1, ACTA2, and C-type 1 mannose receptor (CD206, a macrophage marker), highlighting the role of SGLT2 inhibition in mitigating CaOx stone formation and associated renal damage.

In human proximal tubular cells (HK-2), SGLT2 silencing resulted in reduced expression of OPN and CD44, which are upregulated by high glucose levels. The reduction in CD44, which is particularly involved in crystal adhesion, led to decreased attachment of CaOx crystals to the cells [42]. Additionally, the decrease in OPN, a key protein in regulating crystal formation and aggregation, supports the role of SGLT2 inhibition in reducing CaOx stone formation.

These findings underscore that SGLT2 inhibition specifically targets CaOx stone formation and related inflammation in the renal tubules. Although the primary focus has been on CaOx stones, the potential for SGLT2 inhibitors to impact other calcium-containing stones, such as calcium phosphate stones, which can serve as a nidus for CaOx stone development, is promising. The broader implications of these results highlight the need for further research to fully explore the therapeutic potential of SGLT2 inhibitors in managing various types of kidney stones.

## 4. Novel Treatments Based on the Research Results of SGLT2 Inhibitors for Urolithiasis

Recent studies have highlighted the potential of SGLT2 inhibitors not only for managing diabetes and kidney disease but also for influencing urinary stone formation. Emerging evidence suggests that these inhibitors can alter urinary risk factors for stone formation by changing urinary chemistry in general. Based on these findings, novel treatment strategies could include combination therapy, where SGLT2 inhibitors are used alongside agents that specifically target lithogenic pathways, potentially reducing the risk of both calcium and uric acid stones. Personalized treatment strategies could also be developed, utilizing SGLT2 inhibitors as a first-line therapy for individuals with a history of UL, particularly in those with diabetes and/or CKD. Additionally, the prophylactic use of SGLT2 inhibitors in populations at high risk for recurrent stones warrants investigation. This approach would utilize their renal protective effects while closely monitoring for any increased risk of UTIs, along with other side effects associated with SGLT2 inhibitors. Moreover, clinical trials should focus on the long-term effects of SGLT2 inhibitors on stone recurrence rates and their influence on urinary composition, with an emphasis on identifying optimal dosing and treatment duration. These innovative approaches underscore the need for further research to fully understand the role of SGLT2 inhibitors in managing UL and to establish guidelines for their use in clinical practice.

## 5. Conclusions

In conclusion, UL has become more common worldwide, partly due to rising rates of CRM diseases, which are bidirectionally related to stone formation. SGLT2 inhibitors may help reduce UL risk, particularly in patients with diabetes. However, these inhibitors also pose a potential risk for increased uric acid stone formation. The SWEETSTONE trial, focusing on patients who form stones, will further explore the effectiveness of SGLT2 inhibitors in preventing recurrent kidney stones and their impact on urinary chemistry [60]. Understanding the effects of these medications is key to optimizing treatment and preventing UL complications.

## Data Availability

No new data were created or analyzed in this study.
